# Assessing the performance of the Caregiver Reported Early Development Instruments (CREDI) in rural India

**DOI:** 10.1111/nyas.14543

**Published:** 2020-12-30

**Authors:** Harold Alderman, Jed Friedman, Paula Ganga, Mohini Kak, Marta Rubio‐Codina

**Affiliations:** ^1^ International Food Policy Research Institute Washington DC; ^2^ World Bank Washington DC; ^3^ Department of Political Science Columbia University New York New York; ^4^ World Bank, South Asia Health Washington DC; ^5^ Inter‐American Development Bank, Social Protection and Health Washington DC

**Keywords:** early child development, field assessments, low‐income settings, concurrent validity

## Abstract

Although many education and health programs aim to improve early childhood development, it is challenging to assess developmental levels of infants and small children through large household surveys. The Caregiver Reported Early Development Instruments (CREDI) has been proposed as an adaptable, practical, and low‐cost instrument for measuring the developmental status of children under 3 years of age at scale, as it is relatively short and collected by caregiver report. This study employed the CREDI to measure the development of a sample of 994 children ages 22–35 months in rural India and compared the results to those obtained using the Bayley Scales of Infant and Toddler Development (Bayley‐III), a reliable and widely used instrument, albeit one not always suited to large‐scale data collection efforts given its length, cost, and complexity of administration. The CREDI validation exercise showed that caregivers can provide assessments in keeping with the more interactive (hence more time‐consuming and training‐intensive) Bayley‐III instrument. Noteworthy, there was no indication that concordance of the instruments differed by education of the caregiver. This is important as it points to alternate feasible tools to measure child development outcomes through large‐scale surveys.

## Graphical abstract

The Caregiver Reported Early Development Instruments (CREDI) has been proposed as an adaptable, practical, and low‐cost tool for measuring the developmental status of children under 3 years of age at scale, as it is relatively short and collected by caregiver report. This study employed the CREDI to measure the development of a sample of 994 children ages 22–35 months in rural India and compared the results to those obtained using the Bayley Scales of Infant and Toddler Development, a reliable and widely used instrument.

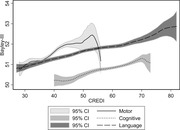

## Introduction

Billions of dollars have been invested in a range of interventions to improve nurturing care. For example, the World Bank and the Inter‐American Development Bank have invested US$5 B combined in early childhood development (ECD) since 2000 across every region of the developing world.^1^ It is hoped that such investments can close long‐term equity gaps in the productivity and earnings of beneficiaries.^2^, ^3^, ^4^ Given the potential importance of such programs as well as the amount invested, there is a need for rigorous evaluation, especially to assess the impact of interventions at scale^5^, ^6^ and to monitor any resulting progress in developmental outcomes at the population level.^7^

These evaluation efforts require instruments to measure ECD that are feasible for use in a wide range of survey conditions, particularly for children under the age of 3 years.^1^, ^7^, ^8^ However, many of the frequently used diagnostic instruments, such as the Bayley Scales of Infant Development,^9^ while reliable^8^, ^10^ and sensitive to differences due to interventions,^11^, ^12^, ^13^ are designed for use by clinically trained professionals in specific contexts—primarily high‐income and westernized^14^—and are difficult and expensive to adapt to field settings in low‐income environments.^15^ Moreover, most of these tests are proprietary, involving expensive test kits and administration fees (copyrights), and their administration is long and requires the presence of the child—all of which makes them impractical for use at large scale.^16^

There is, thus, a need for tools that assess young children's development that are reliable, valid, adaptable, and feasible for use at scale, both for program evaluation and population monitoring. One such instrument is the Caregiver Reported Early Development Instruments (CREDI), a relatively new, open source instrument for assessing ECD outcomes of children 0–36 months of age in culturally diverse settings.^17^ The CREDI has both a short form, designed for large‐scale multipurpose surveys and population‐level monitoring, as well as a long form intended for research and evaluation. The long form of the CREDI was used in this study. Both forms differ from other instruments often used in that they rely entirely on caregiver report and were specifically designed for administration as part of household surveys in low‐resourced areas in a broad array of culturally diverse settings. To date, the CREDI has been piloted in 17 countries.^18^

The reliance on caregiver response is clearly an advantage in terms of ease of implementation compared with direct assessment of children, offering more flexibility on the time and place of administration and the tester profile, as well as substantially reducing test training and administration times and requirements. But these gains are only practical if the information obtained is deemed reliable and valid. In the process of piloting the CREDI, researchers have verified that the instrument had the same relation with a latent development construct whether the sample came from a low‐income country or a high‐income setting and also validated the instrument against other instruments commonly used for measuring child development.^18^ For example, an exercise in Brazil ascertained its concurrent validity with a directly administered measure, the Inter‐American Development Bank Regional Project on Child Development Indicators (PRIDI).^19^, ^20^ Similarly, a study in Tanzania compared the cognitive scale, which also includes language items, to the third edition of the Bayley Scales, the Bayley‐III.^15^, ^21^ A recent review of 27 tools for early child development that covered at least three domains rated the CREDI highly in regard to validity and reliability.^22^

The current study, undertaken in two low‐income, mostly rural, districts in Madhya Pradesh, India, adds additional evidence of the reliability and validity of the CREDI by comparing results from the long form of the CREDI to those observed using the Bayley‐III, often used as a standard to which other instruments have been compared.^16^ As in previous studies, we ascertain the internal consistency of both tests, as well as the concurrence of the caregiver observations given by the CREDI with the direct observation of the child abilities obtained from the Bayley‐III, focusing on cognitive, language, and fine motor development. We not only investigate the covariation of the CREDI and the Bayley‐III with adjustments for age, as in previous studies, but also explore correlations after controlling for the common role of socioeconomic covariates. We expect that the same socioeconomic conditions will be associated with both instruments and conjecture that, after controlling for these common socioeconomic factors, both measures will still indicate similar patterns of child development. We further pay attention to whether the relative performance of the CREDI differs by the education of the caregiver and other characteristics that may affect the caregiver response. This is important for understanding the determinants of developmental heterogeneity observed within a sample population. Finally, we assess whether the CREDI conveys additional information on the relationship of cognitive development to nutrition and care indicators not identified with the Bayley‐III instrument. Through this analysis, we hope to contribute to establishing the reliability and validity of a more practical—namely, convenient to use—tool.

## Methods

### Study setting

The research was undertaken in the context of the endline survey of a Cluster Randomized Control Trial (CRCT) that evaluated the impacts on child development of the expansion of daycare services provided by the Indian Integrated Child Development Services (ICDS) in the districts of Dhar and Singrauli in Madhya Pradesh.^23^ These districts occupy the 32nd and 26th wealth percentiles, respectively, when ranking from poorest to wealthiest, based on the India's National Family Health Survey (NFHS‐4), which is representative at the district level.^24^ The study design was a repeated cross section of children 18–42 months. A baseline survey was undertaken in September–December 2014 for the purposes of the CRCT and the endline survey was completed between January and February 2018. Balance between the treatment and control groups was verified using the baseline data, including balance in ECD outcomes, which were assessed using the Ages and Stages Questionnaire (third edition, ASQ‐3).^25^

Both the baseline and endline samples were designed to be representative of all households in the respective districts with at least one child under 5 years of age, and study size was based on power calculations for the CRCT. As per the CRCT study protocols, the sample included 200 community‐level clusters divided equally between the treatment and control communities. In each community, 15 children were randomly selected from a listing of all children aged 18–42 months residing within a center's catchment area and, therefore, eligible for the program. As a few communities had fewer than 15 children in the age bracket, the endline survey was completed with 2856 households. The full survey, including the long form of the CREDI, was administered to the primary caregivers of the target children (referred to as *index children* hereafter) in each household. The Bayley‐III was administered to the subset of index children who were 22–35 months of age at endline since the maximum age for which the CREDI is designed is 36 months. All told, the analysis sample for the purpose of comparing the CREDI and the Bayley‐III are 994 index children falling within the age range of 22–35 months.

The initial IRB approval by IFPRI's IRB (# 00005121) was amended before endline data collection to accommodate the inclusion of the administration of the CREDI and the Bayley‐III (# 00007490). Caregivers provided written agreement to undertake the data collection. The trial, Making Integration the Operative Concept in the Indian Integrated Child Development Services, was registered at the AEA RTC Registry as AEARTCR‐0000967. Written consent for trial participation was also obtained from caregivers.

### Instruments and training

As indicated, the study compares assessments of child development using the long form of the CREDI (hereafter, referred to simply as the CREDI) with results using the Bayley‐III, which is often considered the gold standard or, at least, one of the most reliable instruments for assessing the development of very young children (under 42 months).^8^, ^10^

There is a total of 109 questions in the long form arranged in increasing order of difficulty. However, the number of responses for the CREDI depends on the child's age and development; there is a different starting point for various age groups and then the interview continues until there are five consecutive “no” answers. CREDI scores were generated by processing the caregiver responses in the software program R via the *credi* package following the instructions of the CREDI team that developed the measure.^26^ The *credi* package calculates scores for each subcategory (motor, cognitive, language, and socioemotional) as well as an overall development score. The Bayley‐III measures cognition, receptive language, expressive language, fine motor, and gross motor development by direct observation of the child's performance in a series of tester‐administered items, arranged in increasing order of difficulty. Basal and ceiling rules determine the number of items to administer to each child.

The Bayley‐III uses the Greenspan Social‐Emotional Growth Chart for the assessment of socioemotional development, which is collected by caregiver report.^27^ However, in the current study, we did not collect the Bayley‐III socioemotional scale since a comparison with the CREDI socioemotional domain would only compare two caregiver reports, an undertaking that was not deemed germane to the task at hand. Instead, we preferred to minimize respondent's fatigue and optimize testing time. Similarly, we did not assess gross motor development with the Bayley‐III because of time and logistical constraints. The gross motor scale requires the use of certain materials (e.g., standardized steps not provided by the publisher), which were difficult to transport in the relatively remote study setting. Thus, for the purpose of the current analysis, only the cognitive, language (both receptive language and expressive language), and fine motor scales of the Bayley‐III were used.

Both the CREDI and Bayley‐III instruments were translated into Hindi and back translated. Piloting indicated small differences in the dialects of Hindi in the two districts and minor accommodations were made to reflect these. RehabInsights, a firm with prior experience adapting and implementing the Bayley‐III in the Indian context, was responsible for the adaptation of this instrument to local conditions and to train a separate team of dedicated testers (“testers” henceforth). Many pictures in the standard instrument were adapted after pretesting as the originals were not familiar to residents in the study site. For example, a banana was substituted for a strawberry, bathing was substituted for swimming, and a samosa was substituted for a cake. A complete list of adaptations is available on request.

The CREDI was administered during household interviews conducted by staff of the Oxford Policy Management Ltd. Thirty‐five interviewers were trained for 10 days on the main survey instrument, which included the CREDI. The Bayley‐III was conducted at the day care center (the Anganwadi center) of the ICDS, wherever possible. If the index child was not available for the test at the Anganwadi center, testing was done at the child's home (and this was controlled for in the analysis). Twenty percent of tests were undertaken in this manner. The protocol was to conduct both tests on the same day whenever possible. The fact that one test was with the caregiver made this feasible.

Bayley‐III testers were provided an extensive 6‐week training from November 27, 2017 to January 10, 2018. The training period was divided into two parts: 2 weeks of theory training were followed by 1 week of practical training and 3 weeks of field practice. Each tester had a chance to practice on a minimum of 20 children. During these practice sessions, interobserver reliabilities were calculated among the testers and supervisors. While the training was conducted for a total of 18 testers, in the end, 10 were selected based on their performance to continue with data collection.

We seek to assess the association of the CREDI instrument and other aspects of childcare and child outcomes. Thus, additional information collected during the household endline included data on anthropometry for the index child, collected following standard WHO protocols, as well as information on service provision from the ICDS. Caregiver characteristics, including educational attainment, were also collected. Maternal symptoms of depression were measured using the Center for Epidemiologic Studies Short Depression Scale (CESD‐10), an adaptation of the 20‐question scale in common use in a range of settings, including rural India.^28^, ^29^ We categorized mothers with high depressive symptoms if their total score was of 10 or higher (out of a total of 30) on the CESD‐10 and with low depressive symptoms, otherwise.^29^ In addition, the survey collected data on household assets and a household wealth index was constructed using principal component analysis.^30^ Finally, the survey collected information on the Family Care Indicators (FCI), a commonly used measure of the quality of the home environment.^31^

### Analytic framework

The CREDI instrument is designed for children from birth up to 36 months. However, the CRCT required that the index child be old enough to have spent time in day care. Since many sampled clusters had relatively few children in the 22–35 months age bracket, which is included in CREDI coverage, 24.7% of the index children were between 36 and 42 months.

Given that the performance of the CREDI at the boundary of ages for which it is designed is relevant to program evaluations, where ages are often recorded with error, we included all children (22–42 months of age) in the wider survey of 2856 households in Figures S1–S3 (online only), which illustrate possible ceiling effects in the assessed CREDI domains. All other figures and tables in this report are for the subsample of 994 children aged 22–35 months included in the direct comparison. Figures S1–S3 (online only) show that there were ceiling effects for only a modest proportion of items for children older than 36 months, and for very few items for younger children. As expected, the proportion of positive caregiver responses declines as item difficulty increases (those in the lower half of Fig. S1, online only) for children of all ages.

In our analysis, we used the R software provided by CREDI to calculate a *raw scaled* (*factor*) *score* for each domain. Raw scores from both instruments were then internally age‐standardized using age‐conditional means and standard deviations (SDs) following the nonparametric method proposed elsewhere^16^ with two modifications: we used age in months (instead of in days) and we did not remove interviewers/testers’ effects before standardizing. The R package also provides a “norm‐referenced standardized score (Z‐score)” for each domain. Specifically, the referencing subtracts the average raw score of children in a global reference population of the same age in months from the observed raw score and then divide the difference by the age‐specific SD. A Z‐score of 0 thus means that the child has exactly the same score on that particular domain as the average same‐age child in the CREDI reference population. A score of “−1” means that the child's raw score is 1 SD below the same‐age average of the reference population.

The Bayley‐III also provides norm‐referenced scores.^32^ However, to utilize these for motor development, it is necessary to have observations on both gross and fine motor development. Thus, the majority of the results reported in the main body of this study use results that are referenced by survey age conditional means; for comparison, we report one key table as an appendix using the global norm‐referenced scores for cognitive and language domains based on the R software package for the CREDI and the Bayley‐III administration manual.

We first investigated the reliability of the measures by exploring the internal consistency of each tests’ domain using Cronbach's alpha (α) for the entire sample and separately by maternal education (no education versus some education). We stratified by education as a possible key mediating factor for the CREDI, but not the Bayley‐III, is the education level of the respondent. However, any comparison of scores across subgroups, such as educational categories, is conditional on the assumption that groups view the survey items the same; that is, the measurement structure of the latent cognitive domain is “invariant.”^33^ Thus, we applied a structural equation modeling approach for latent index construction to the CREDI cognitive, language, and motor domains to assess measurement invariance.

We next investigated validity. We assessed the performance and covariation of the developmental scores from the two instruments in a variety of ways. We started by conducting graphical descriptive analysis on the scores from the two instruments, using nonparametric regressions. To account for the possibility that any observed correlation would reflect the expected improvement of unadjusted scores as a child ages, we present graphical analysis with raw scores as well as with age adjusted scores. We then computed the concurrence between scores by domain using Pearson correlations (*r*) on both raw (unadjusted) and internally age‐standardized scores (adjusted).

As such concurrence may be driven by the fact that both indicators are strongly associated with common factors, we subsequently decompose the association in scores to covariation because of observed characteristics and covariation in residuals. To do this, the analysis first investigated the conditional associations in each test between the internally age‐standardized aggregate development scores and predictors, such as maternal education and household asset wealth, which have been shown in numerous previous studies to be significantly related to child development.^34^ We then controlled for the influences of these predictors by estimating the residuals from the scores regressed on socioeconomic covariates. We investigated the cross‐test correlations of these residuals, which provide evidence of the concurrence of the information on child development in each domain of each test net of the linear influence of key socioeconomic factors. As the education level of the respondent might influence the reliability of her assessment, we investigated the stability of the associations between the two scales across different levels of caregiver formal education.

Finally, we related the developmental scores to child height‐for‐age (HAZ) as well as a measure of home stimulation environment—two outcomes that have been shown in numerous settings to be related to assessments of child development.^6^, ^29^ The strength of association of each development score to these outcomes was assessed.

## Results

Table 1 conveys the mean values for all characteristics of the children and caretakers in the 994 children used in the analysis sample. We report the mean for the raw scores as well as the global norm referenced scores but do not report the locally normed scores as they are centered at 0 by construction. Thirteen percent of the Bayley‐III assessments were observed by a study supervisor and only 1% of the assessments involved a child break in which the child had something to drink. Both factors may influence performance in the Bayley‐III and consequently are controlled for in subsequent analysis. The mean age of an assessed child is almost 29 months, and 51% of the children are boys. The asset wealth score of the household is assigned to a quartile indicator, and as expected, each quartile indicator contains roughly 25% (ranging from 23% to 27%) of the sample. The educational attainment of the mothers in the study sample ranges from no formal education to post‐secondary completion. Given the rural concentration of the sample, the education distribution skews toward relatively low levels of educational attainment with 59% of all mothers reporting primary level education completed or less. Only 11% of mothers report secondary attainment or greater.

**Table 1:** Test scores and characteristics of children in the study sample and their caregivers

	Mean/proportion	Standard deviation
*CREDI scores*		
CREDI population standardized overall	−0.70	1.01
CREDI population standardized cognitive	−0.82	1.21
CREDI population standardized motor	−0.61	1.18
CREDI population standardized language	−0.60	0.97
CREDI raw score overall	51.34	0.88
CREDI raw score cognitive	50.80	0.87
CREDI raw score motor	51.49	1.06
CREDI raw score language	51.60	1.00
*Bayley‐III scores*		
Bayley‐III population standardized cognitive	83.34	14.28
Bayley‐III population standardized language	86.20	17.75
Bayley‐III score cognitive	58.59	6.08
Bayley‐III score fine motor	38.56	4.25
Bayley‐III score language	50.09	11.05
Bayley‐III taken at home	0.20	−
Was the test observed?	0.13	−
Were there breaks?	0.01	−
*Child and mother characteristics*		
Child age (months)	28.92	3.47
Treatment Wealth index	0.50	−
Bottom wealth quartile	0.23	−
2nd wealth quartile	0.27	−
3rd wealth quartile	0.25	−
Top wealth quartile	0.24	−
Child gender (male, *n* = 1)	0.51	−
Mother's depression	0.17	0.37
Mother's education		
No education	0.39	−
Primary (less than 5 years)	0.20	−
Middle (6−8 years)	0.17	−
Higher (9−10 years)	0.10	−
Secondary and above (more than 11 years)	0.11	−
Observations	994	

note: The table includes the CREDI and Bayley‐III raw scores. The wealth index is generated from a principal components analysis (PCA) of underlying household assets. The variables “Was the test observed?” and “Were there breaks?” account for whether an interviewer/tester was present during the survey and whether the child took a break during the survey. For the categorical variables “Wealth index” and “Mother's education,” we report the proportion for each category.

**Figure 1:**
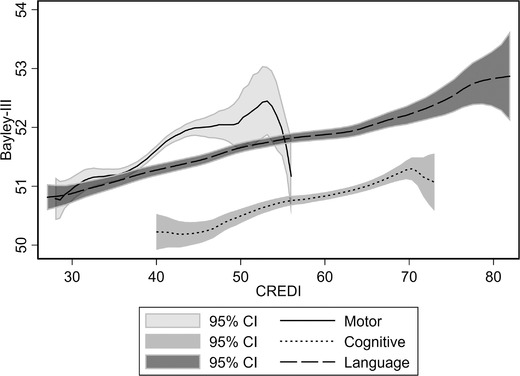
Nonparametric relation between CREDI and Bayley‐III's raw scores. The Pearson correlation coefficient is 0.277 for cognitive scores, 0.310 for motor scores, and 0.405 for language scores.

**Figure 2:**
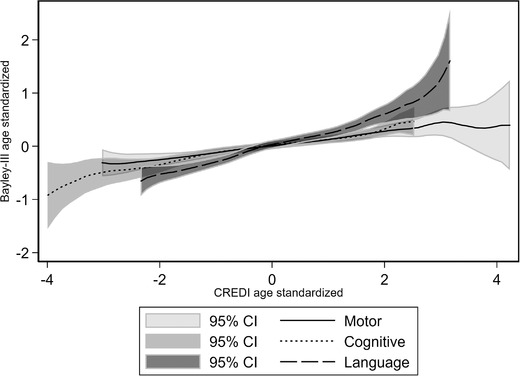
Nonparametric relation between CREDI and Bayley‐III's locally age‐adjusted scores. The Pearson correlation coefficient is 0.212 for cognitive scores, 0.199 for motor scores, and 0.332 for language scores.

Figure 1 relays the nonparametric local polynomial association between the CREDI and Bayley‐III raw scores, separately for the language, cognitive, and motor domains. These were constructed using the lpolyci nonparametric regression command in STATA with the standard kernel default. The Pearson's correlation coefficient in developmental scores is estimated to be 0.40 for the language domain, 0.28 for the cognitive domain, and 0.31 for the motor domain. Figure 1 indicates a generally positive association throughout the distribution of CREDI scores, with the possible exception of the upper range of CREDI scores for motor and cognitive development (but not language) where the association with Bayley‐III appears to weaken. Figure 2 again depicts the nonparametric associations between the two measures, but now the scores have been age standardized. The slope of the associations of scores between the CREDI and the Bayley‐III do not differ by domain. The correlation coefficients between the two scores are still precisely estimated and positive, however, lower in magnitude at 0.21 for the cognitive domain, 0.33 for the language domain, and 0.20 for the motor domain.

Table 2 indicates the internal consistency of the scores for each domain for both the CREDI and the Bayley‐III using Cronbach's alpha (α). The internal consistency of any domain either approaches or exceeds the conventional cutoff of 0.7, indicating a degree of consistency^35^ and is in accordance with that observed in similar studies.^15^ Moreover, estimated internal consistency in the CREDI was no different for uneducated compared with educated mothers. This is notable since the CREDI is a caregiver‐reported instrument for which interpretation and recall of children's abilities may be influenced by the education level of the respondent. There are also no differences in consistency by the age of the child (results not shown).

**Table 2:** Internal consistency of the CREDI and Bayley‐III in the sample (Cronbach's alpha)

		Cronbach's alpha by mother's education		
	Cronbach's alpha	No education	Any education	Number of items
CREDI cognitive	0.8013	0.8089	0.7901	25
CREDI language	0.7547	0.8915	0.8901	36
CREDI fine motor	0.7741	0.7825	0.7677	23
Bayley‐III cognitive	0.9088	0.9114	0.9100	43
Bayley‐III language	0.9409	0.9317	0.9455	69
Bayley‐III fine motor	0.8958	0.8951	0.8978	32

As education is an important mediating factor, we assessed metric invariance with respect to the education group of the caregiver and found that only the cognitive domain exhibits metric invariance (*P* < 0.01). However, the requirements for testing for measurement or metric invariance are difficult to meet, and strict forms of measurement invariance rarely hold.^33^ The approach employed tests for invariant factor loadings on the individual question items; thus, the test is sensitive to the number of individual items. Since the CREDI has numerous individual items, we also explored metric invariance with respect to a random subset of 10 individual items in the language and motor domains. When we do so, we observe metric invariance for the motor domain (*P* < 0.05) but not language. Thus, there is some evidence for measurement invariance in the CREDI, at least the cognitive and motor domains, and that differences in scores across education group represent real differences in the latent construct.

Various socioeconomic factors are widely recognized to affect child development. Table 3 investigates the relationship between development and key socioeconomic covariates for both the CREDI and the Bayley‐III. As the scores are standardized by age and normalized, each coefficient expresses the change in SDs of the score associated with a unit change in the characteristic. For both measures, all developmental domains—cognitive, motor, and language—are positively associated with wealth and caregiver education, associations that are generally statistically significant. For example, standardized scores for the wealthiest quartile in all domains are at least 0.6 SD higher than for the poor, and in the case of Bayley‐III language more than 1 SD higher. Children whose mothers had 6–8 years of education—that is, early middle school level—had scores in the neighborhood of 0.2 SD higher than those whose mothers had not gone to school. While the point estimates (and statistical precision) were not larger when the mother had more secondary education, the point estimates were larger for the children whose mothers had schooling beyond secondary. Most germane to the question of CREDI performance vis‐à‐vis the Bayley‐III, coefficients for measures of education and assets are similar between the two instruments, except for gender. In the CREDI cognitive domain, boys scored significantly lower than girls, while the opposite was the case for the Bayley‐III.

**Table 3:** Conditional associations between CREDI or Bayley‐III locally age‐standardized scores and covariates

	CREDI cognitive	Bayley‐III cognitive	CREDI motor	Bayley‐III fine motor	CREDI language	Bayley‐III language
Child age (months)	0.004	0.028***	0.008	0.009	0.007	0.021***
	(0.007)	(0.008)	(0.008)	(0.008)	(0.008)	(0.008)
Child gender (male, *n* = 1)	−0.120**	0.190***	−0.071	0.048	−0.155***	−0.026
	(0.051)	(0.056)	(0.053)	(0.059)	(0.053)	(0.056)
Household wealth						
Bottom wealth quartile	−	−	−	−	−	−
2nd wealth quartile	0.168**	0.098	0.153**	0.167**	0.099	0.268***
	(0.072)	(0.079)	(0.075)	(0.083)	(0.075)	(0.079)
3rd quartile	0.228***	0.243***	0.232***	0.215**	0.286***	0.481***
	(0.075)	(0.082)	(0.078)	(0.086)	(0.079)	(0.082)
4th quartile	0.572***	0.678***	0.547***	0.679***	0.681***	1.034***
	(0.078)	(0.087)	(0.082)	(0.091)	(0.082)	(0.087)
Maternal education						
No school	−	−	−	−	−	−
Primary (less than 5 years)	0.127*	0.181**	0.064	0.129	0.069	0.236***
	(0.070)	(0.077)	(0.073)	(0.080)	(0.073)	(0.077)
Middle (6−8 years)	0.174**	0.133*	0.225***	0.222***	0.192**	0.210***
	(0.072)	(0.080)	(0.075)	(0.084)	(0.076)	(0.080)
Higher (9−10 years)	0.205**	0.103	0.262***	0.102	0.104	0.112
	(0.088)	(0.097)	(0.092)	(0.102)	(0.092)	(0.097)
Secondary and above (more than 11 years)	0.345***	0.261***	0.337***	0.214**	0.276***	0.341***
	(0.089)	(0.099)	(0.093)	(0.104)	(0.094)	(0.099)
Was the test home?	0.113*	0.138*	0.118*	0.093	0.086	0.038
	(0.065)	(0.073)	(0.068)	(0.077)	(0.069)	(0.073)
Was the test observed?		−0.224***		−0.177**		−0.213**
		(0.083)		(0.087)		(0.083)
Were there breaks?		−0.484*		−0.394		−0.503*
		(0.257)		(0.270)		(0.257)
Constant	−0.515*	−0.840***	−0.486*	−0.285	−0.682**	−0.849***
	(0.278)	(0.268)	(0.290)	(0.281)	(0.291)	(0.268)
Observations	994	994	994	994	994	994
Adjusted *R* ^2^	0.392	0.204	0.331	0.153	0.326	0.239

note: All coefficients derive from ordinary least squares (OLS) regressions with standard errors clustered at the survey enumeration (village) level. CREDI and Bayley‐III scores are locally age standardized. Age is added in the analysis to correct for any residual variance. The wealth index is generated from a PCA of underlying household assets. The variables “Was the test observed?” and “Were there breaks?” account for whether an interviewer/tester was present during the survey and whether the child took a break during the survey. All regressions include interviewer/tester fixed effects. Standard errors in parentheses.

^*^ *P* < 0.10; ^**^ *P* < 0.05; ^***^ *P* < 0.01.

The results in the first three rows of Table 4 indicate correlations of residuals after regressing locally age‐standardized scores on control variables. Correlations with residuals using raw scores of the CREDI and the Bayley‐III are also included in parenthesis for comparison. The first set include only controls for tester/interviewer as well as an indicator for the few tests that were paused to allow the child to rest. This parallels a similar analysis in Tanzania.^15^ The next set of columns relay the correlations of residuals from regressions that sequentially add the caregiver's education and subsequently the household socioeconomic quartile. As expected, the correlations decline as additional adjustments for common determinants are included; adding both education and socioeconomic quartile reduces the correlations by approximately 25%. The correlations with all the adjustments are, nevertheless, significantly different from zero (and positive). The relatively smaller correlations for motor development may reflect the fact that the CREDI motor scale includes both gross and fine motor items while only the Bayley‐III fine motor scale was collected.

**Table 4:** Correlations of Bayley‐III Residuals with CREDI Residuals (N = 994)

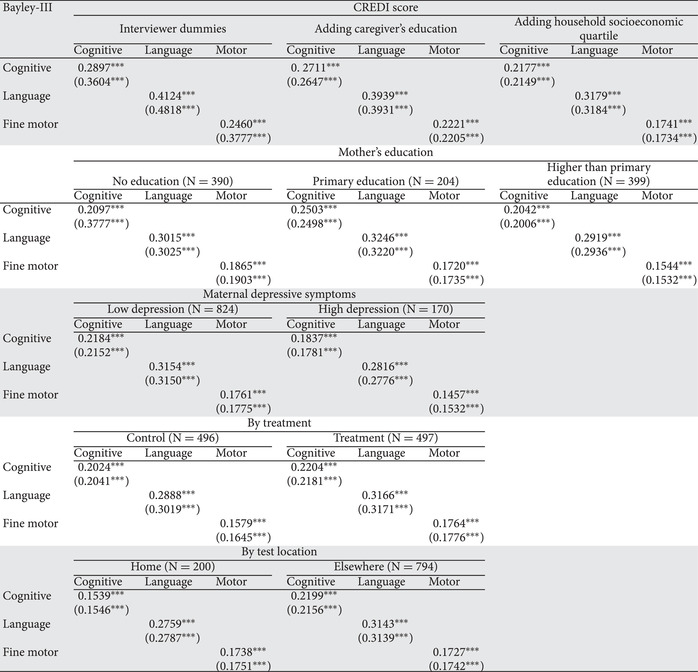

note: All coefficients derive from OLS regressions with standard errors clustered at the survey enumeration (village) level. CREDI and Bayley‐III scores are locally age standardized. Age is added in the analysis to correct for any residual variance. The wealth index is generated from a PCA of underlying household assets. The variables “Was the test observed?” and “Were there breaks?” account for whether an interviewer/tester was present during the survey and whether the child took a break during the survey. All regressions include interviewer/tester fixed effects. Standard errors in parentheses.

^***^ *P* < 0.01.

The next three rows of Table 4 reveal that the correlations of caregiver‐reported cognitive, language, and motor development, and the Bayley‐III assessments do not differ appreciably by level of education after controlling for child and caregiver characteristics. This addresses a concern that less educated caregivers might report the activities of their child in a manner that was less in conformity with independent observations than are observations by those with more education. In particular, there is no support to either the view that uneducated caregivers might be less accurate in reporting the skills of their child relative to the Bayley‐III or conversely that the most educated caregivers might see skills in their child that are less apparent to the staff administrating the Bayley‐III.

As indicated further in Table 4, however, mothers who report relatively high numbers of symptoms of depression seem to report skills that are less in accord with the Bayley‐III results than those reported by caregivers with fewer symptoms. For example, the correlation of cognitive scores as reported by mothers with low depressive symptoms with the Bayley‐III cognitive scores is 19% larger than it is for mothers with more depressive symptoms.

It is possible that greater contact with service providers, as occurred in the treatment group of the CRCT, might make caregivers more aware of development milestones and, thus, improve their ability to assess the development of their child. The next row of Table 4 indicates that there is only a small difference in the association of the results in the two indicators by treatment status in the CRCT. That is, caregivers of participants in the day care program report development of their child that is only slightly more in concordance with the results in the Bayley‐III. Finally, the bottom panel of the table shows that there are slight differences in the correlations based on where the Bayley‐III test took place. The correlations of residuals are quite similar to those reported in Table 4 when the global population reference groups are used to standardize results instead of when age‐standardizing using the survey data (Table S1, online only).

Table 5 presents regressions that explore the joint association of both the CREDI and the Bayley‐III with two variables widely believed to be strongly associated with a child's level of development, and often used as proxies: early life nutrition and disease exposure, as summarized in the HAZ score, and the home environment, as measured by the FCI. Although no direction of causality is implied by the regressions, they indicate that information in both the CREDI and Bayley‐III scores is significantly and, at times, independently associated with these two measures. Interestingly, the FCI is positively related to both the Bayley‐III and CREDI language domains (and not other domains), indicated by the fact that both the coefficient of the CREDI and the Bayley‐III language domains are significant at *P* < 0.01 in the regression for FCI. While the conditional association is greater in magnitude for the CREDI than the Bayley‐III, the two associations are not statistically significantly different from each other. This suggests that there may be independent elements in the two language domains that identify complementary but distinct aspects of language development. Regarding the HAZ Z‐score, it is the CREDI cognitive domain and the Bayley‐III gross motor domain that are positively related to HAZ. This suggests that, while the CREDI motor domain adds little over the (more associated) Bayley‐III motor domain with regard to predicting HAZ, it is the opposite case with regard to the cognitive domain—the conditional covariation of HAZ loads onto the CREDI cognitive score and not the Bayley‐III. We take this as suggestive evidence that the Bayley‐III and CREDI index may measure somewhat distinct elements of child development, rather than entirely the same construct.

**Table 5:** Conditional associations between CREDI and Bayley‐III's scores and other developmental measures

	Height for age Z‐score	FCI score
CREDI cognitive	0.219**	0.074
	(0.093)	(0.050)
CREDI motor	−0.120	−0.026
	(0.080)	(0.043)
CREDI language	0.044	0.160***
	(0.074)	(0.040)
Bayley‐III cognitive	0.004	−0.015
	(0.054)	(0.029)
Bayley‐III motor	0.227***	0.017
	(0.051)	(0.027)
Bayley‐III language	0.073	0.080***
	(0.055)	(0.030)
Child age (months)	−0.018*	0.006
	(0.011)	(0.006)
Child gender (male, *n* = 1)	−0.007	0.036
	(0.076)	(0.041)
Household wealth		
Bottom wealth quartile	−	−
2nd wealth quartile	0.001	0.106*
	(0.107)	(0.058)
3rd wealth quartile	0.067	0.150**
	(0.114)	(0.062)
Top wealth quartile	0.361***	0.473***
	(0.127)	(0.069)
Maternal education		
No school	−	−
Primary (less than 5 years)	0.132	0.043
	(0.103)	(0.056)
Middle (6−8 years)	0.018	0.040
	(0.108)	(0.058)
Higher (9−10 years)	0.112	0.044
	(0.131)	(0.071)
Secondary and above (more than 11 years)	0.370***	0.397***
	(0.134)	(0.073)
Constant	−1.811***	0.393
	(0.451)	(0.244)
Observations	993	994
Adjusted *R* ^2^	0.134	0.454

note: All CREDI and BAYLEY‐III scores are locally age standardized. The wealth index is generated from a PCA of underlying household assets. All regressions include interviewer/observer fixed effects, test location, and information on breaks. Standard errors in parentheses.

^*^ *P* < 0.10.; ^**^ *P* < 0.05; ^***^ *P* < 0.01.

## Discussion

Results from this application of the CREDI indicate that the instrument appears to perform well with regard to a recognized standard in rural northern India. This is in line with the evidence from other studies^18^ and supports the view that the CREDI is useful within a range of settings, including low income/low education communities. In many respects, the correlations that removed the common influences of education and wealth covariates, both of which are associated with measures of child development, are particularly relevant for this assessment. The results show that after adjusting for underlying determinants, the information in individual development scores in the CREDI remains significantly correlated with information provided by the more resource‐intensive Bayley‐III. Furthermore, the results provide supportive evidence indicating that both measures reflect the child's environment in a similar but not identical manner.

This conclusion is also supported by the fact that household wealth and caregiver education are associated with age standardized CREDI scores over the three domains of cognition, language, and motor development in accord with international experience.^18^, ^36^ Moreover, the association with the CREDI is quantitatively close to the association of these covariates with the standardized Bayley‐III scores. That the two measures show concordance reinforces previous evidence that the CREDI is useful for assessing child development in a community,^18^ subject, of course, to the caveat as to inference from the context of a single culture and restricted age coverage. At the same time, the two instruments have independent associations with nutrition and indicators of the quality of the home care environment implies that they provide complementary information and may be most useful in tandem rather than as substitutes.

The reason for the gender difference in the results of these two instruments, however, is not readily apparent. The CREDI results here are more in keeping with past studies using the instrument^18^ than are those for the Bayley‐III, which generally show that girls outperform boys in contrast to the results reported here.^37^, ^38^ The differences may be related to a gender related bias on the part of the Bayley‐III assessors. Alternatively, or additionally, young boys and girls in this region of India may differ in their reticence to perform for the Bayley‐III assessor; such differences in desire to perform have been noted in various settings.^39^, ^40^ The current study, however, was not designed to assess the reasons the CREDI differs from the Bayley‐III on this one pattern. Such gender differences are, nevertheless, an area worthy of future investigation.

The results here also address one potential drawback of the CREDI, that relatively uneducated caregivers might be less likely to provide information that corresponds to the observations of a trained researcher than would their more educated neighbors.^8^ In this study, caregiver (respondent) education does not appear to be a barrier to the validity of information conveyed in self‐reported child development assessments. While the difference in the association with the Bayley‐III with regard to maternal depression is modest in our study, it may reflect such a barrier, and would need to be further investigated in future studies.

We did not have multiple tests of the same child over time, so the study did not add to the evidence that the CREDI is reliable as defined by the within‐subject intertemporal reliability of results. Another limitation of the study is that we are unable to compare the two instruments as indicators of project impact. Although the data were collected as part of a CRCT, neither cognitive nor motor domains were affected by the treatment using the full CREDI sample.^23^ Thus, the comparison provides little insight about whether these two measures are equally capable of capturing effects of an intervention. For example, although we see a small difference in the association of CREDI and Bayley‐III scores by treatment status, neither of these outcomes are influenced by the intervention; the null effect could not be rejected with a level of significance of *P* < 0.10. Therefore, the current study does not address whether the CREDI is as useful for program impact evaluation as other established methods. The fact that we are unable to determine whether the CREDI would be subject to social desirability bias when used in an assessment of a program that aimed to provide center‐based early childcare hints at an important area for future research.

Another drawback is the restricted age range. We cannot say anything for children younger than 22 months, for which the assessment of child development is more complex and the number of easy to use instruments further limited—an area for future investigation. Additionally, future research can assess the longitudinal predictive reliability of the CREDI relative to other caregiver responses, as well as compared with direct professional assessment. As any instrument for assessing child development will be called upon for these tasks, such research will help determine the role of the CREDI as a component of an ECD toolkit. As mentioned, the goal of this study was to assess caregiver response vis a vis direct child assessment. As such, the socioemotional domain was not covered in this paper. It remains, however, a potentially important dimension of the CREDI instrument.

All told, the results suggest that the CREDI may be a suitable alternative to expert assessment in a variety of low‐income contexts and, moreover, may be a useful complement to expert assessments in other studies. Indeed, items in the CREDI have already contributed to the Global Scale for Early Development (GSED), a new effort, currently in development, aimed at generating two globally applicable instruments—that is, internationally standardized and validated instruments—for the assessment of ECD for children under age 3 years at population (short form) and programmatic (long form) levels. Led by the WHO, the GSED group encompasses the harmonization of the CREDI and the instruments and methodologies developed by two other groups: the Young Child Development group and the Global Child Development group.^41^ The instrument has also been used in the design of the Early Child Development Index, which is part of UNICEF's Multiple Indicator Cluster Surveys.

## Conclusion

That the CREDI appears to perform well with regard to a recognized standard, and at a lower cost than that standard, in disparate contexts, suggests that the CREDI may be a suitable alternative to expert assessment in large‐scale surveys in a variety of low‐income contexts. It exhibits adequate validity with respect to the more resource‐intensive Bayley‐III assessment. Given its comparative simplicity, the CREDI can be relatively easily included in environmental studies and as a practical indicator for a range of child‐oriented projects. Furthermore, it has potential to serve as a component for a multidimensional global instrument to assess child development.

The study was funded by the Strategic Impact Evaluation Fund administered by the World Bank as well as POSHAN (Partnerships and Opportunities to Strengthen and Harmonize Actions for Nutrition in India), funded by the Bill & Melinda Gates Foundation and managed by IFPRI. The funders had no role in the analysis. The authors wish to thank Dana McCoy and Gunther Fink for advice on implementing the CREDI, and to Debashis Panda for assistance with the Bayley–III.

## Author contributions

H.A., J.F., and M.R.‐C. conceived the study and shared the writing. M.K. oversaw the field work. P.G. contributed extensively to data analyses.

## Competing interests

The authors declare no competing interests.

## Supporting information


**Supplementary Figure S1**.

**Supplementary Figure S2**.

**Supplementary Figure S3**.

**Supplementary Table S1**. Correlations of Bayley‐III Residuals with CREDI Residuals using global normed scores (N = 994)

## References

[1] Black, M.M. , S.P. Walker , L.C. Fernald , *et al*. 2017. Early childhood development coming of age: science through the life course. Lancet 389: 77–90. 27717614 10.1016/S0140-6736(16)31389-7 PMC5884058

[2] Engle, P.L. , L.C. Fernald , H.H. Alderman , *et al*. 2011. Strategies for reducing inequalities and improving developmental outcomes for young children in low‐income and middle‐income countries. Lancet 378: 1339–1353. 21944378 10.1016/S0140-6736(11)60889-1

[3] Campbell, F. , G. Conti , J.J. Heckman , *et al*. 2014. Early childhood investments substantially boost adult health. Science 343: 1478–1485. 24675955 10.1126/science.1248429 PMC4028126

[4] Gertler, P. , J. Heckman , R. Pinto , *et al*. 2014. Labor market returns to an early childhood stimulation intervention in Jamaica. Science 344: 998–1001. 24876490 10.1126/science.1251178 PMC4574862

[5] Aboud, F.E. , A.K. Yousafzai & M. Nores . 2018. State of the science on implementation research in early child development and future directions. Ann. N.Y. Acad. Sci. 1419: 264–271. 29791728 10.1111/nyas.13722

[6] Britto, P.R. , M. Singh , T. Dua , *et al*. 2018. What implementation evidence matters: scaling‐up nurturing interventions that promote early childhood development. Ann. N.Y. Acad. Sci. 1419: 5–16. 29791739 10.1111/nyas.13720

[7] McCoy, D.C. , M.M. Black , B.B. Daelmans & T. Dua . 2016. Measuring development in children from birth to age 3 at population level. Bernard van Leer Foundation, The Hague, Netherlands. 34–39.

[8] Fernald, L.C. , E. Prado , P. Kariger & A. Raikes . 2017. A Toolkit for Measuring Early Childhood Development in Low and Middle‐Income Countries. Washington, DC: World Bank.

[9] Bayley, N. 1969. Bayley Scales of Infant Development. New York: Psychological Corp.

[10] Frongillo, E.A. , F. Tofail , J.D. Hamadani , *et al*. 2014. Measures and indicators for assessing impact of interventions integrating nutrition, health, and early childhood development. Ann. N.Y. Acad. Sci. 1308: 68–88. 24372533 10.1111/nyas.12319

[11] Hamadani, J.D. , S.N. Huda , F. Khatun & S.M. Grantham‐McGregor . 2006. Psychosocial stimulation improves the development of undernourished children in rural Bangladesh. J. Nutr. 136: 2645–2652. 16988140 10.1093/jn/136.10.2645

[12] Grantham‐McGregor, S.M. , A. Adya , O.P. Attanasio , *et al*. 2020. Group sessions or home visits for early childhood development in India: a cluster RCT. Pediatrics 146: e2020002725. 33148771 10.1542/peds.2020-002725 PMC7786825

[13] Attanasio, O.P. , C. Fernandez , E.O.A. Fitzsimons , *et al*. 2014. Using the infrastructure of a conditional cash transfer program to deliver a scalable integrated early child development program in Colombia: cluster randomized controlled trial. BMJ 349: g5785. 25266222 10.1136/bmj.g5785 PMC4179481

[14] Henrich, J.S.J. & H.A. Norenzayan . 2010. The weirdest people in the world? Behav. Brain Sci. 33: 61–83. 20550733 10.1017/S0140525X0999152X

[15] McCoy, D.C. , C.R. Sudfeld , D.C. Bellinger , *et al*. 2017. Development and validation of an early childhood development scale for use in low‐resourced settings. Popul. Health Metr. 15: 3. 28183307 10.1186/s12963-017-0122-8 PMC5301363

[16] Rubio‐Codina, M. , M. Caridad Araujo , O.P. Attanasio , *et al*. 2016. Concurrent validity and feasibility of short tests currently used to measure early childhood development in large scale studies. PLoS One 11: e0160962. 27548634 10.1371/journal.pone.0160962 PMC4993374

[17] https://sites.sph.harvard.edu/credi/credi-materials/. Accessed October 5, 2020.

[18] McCoy, D.C. & M. Waldman ; CREDI Field Team and Günther Fink . 2018. Measuring early childhood development at a global scale: evidence from the Caregiver‐Reported Early Development Instruments. Early Child. Res. Quart. 45: 58–68.

[19] Altafim, R.P. , D.C. McCoy , A. Brentani , *et al*. 2020. Measuring early childhood development in Brazil: validation of the Caregiver Reported Early Development Instruments (CREDI). J. Pediatr. 96: 65–77. 10.1016/j.jped.2018.07.008 PMC9432122 30102876

[20] Verdisco, A. , J. Thompson & O. Neuschmidt . 2014. PRIDI: urgency and possibility. Inter‐American Development Bank.

[21] Bayley, N. 2006. Bayley scales of infant and toddler development. 3rd ed. Technical Manual. Harcourt Assessment, San Antonio, TX.

[22] Boggs, D. , K.M. Milner , J. Chandna , *et al*. 2019. Rating early child development outcome measurement tools for routine health programme use. Arch. Dis. Child. 104(Suppl. 1): S22–S33. 30885963 10.1136/archdischild-2018-315431 PMC6557219

[23] World Bank . 2018. Impact evaluation of an AWC‐cum‐creche pilot in Madhya Pradesh. Report No: AUS0000253 September.

[24] International Institute for Population Sciences (IIPS) and ICF . 2017. National Family Health Survey (NFHS‐4), 2015–16. Mumbai: IIPS.

[25] Squires, J. , D. Bricker , E. Twombly , *et al*. 2009. Ages & Stages English Questionnaires, Third Edition (ASQ‐3): A Parent‐Completed, Child‐Monitoring System. Baltimore, MD: Paul H. Brookes Publishing Co.

[26] https://cdn1.sph.harvard.edu/wp-content/uploads/sites/2435/2016/05/CREDI-Scoring-Manual-8-Jun-2018.pdf. Accessed Oct. 5, 2020.

[27] Greenspan, S.I. 2004. Greenspan Social‐Emotional Growth Chart: A Screening Questionnaire for Infants and Young Children. San Antonio, TX: Harcourt Assessment.

[28] Radloff, L.S. 1977. The CES‐D scale: a self‐report depression scale for research in the general population. Appl. Psychol. Measure. 1: 385–401.

[29] Nguyen, P.H. , J. Friedman , M. Kak , *et al*. 2018. Maternal depressive symptoms are negatively associated with child growth and development: evidence from rural India. Matern. Child Nutr. 14: e12621. 29770998 10.1111/mcn.12621 PMC6175434

[30] Filmer, D. & L.H. Pritchett . 2001. Estimating wealth effects without expenditure data–or tears: an application to educational enrollments in states of India. Demography 38: 115–132. 11227840 10.1353/dem.2001.0003

[31] Kariger, P. , E.A. Frongillo , P.L. Engle , *et al*. 2012. Indicators of family care for development for use in multicountry surveys. J. Health Popul. Nutr. 30: 472–486. 23304914 10.3329/jhpn.v30i4.13417 PMC3763619

[32] Bayley, N. 2006. Bayley scales of infant and toddler development: administration manual. Harcourt Assessment.

[33] Van De Schoot, R. , P. Schmidt , A. De Beuckelaer , *et al*. 2015. Editorial: measurement invariance. Front. Psychol. 6. 10.3389/fpsyg.2015.01064. PMC4516821 26283995

[34] Fink, G. , D.C. McCoy & A. Yousafzai . 2020. Contextual and socioeconomic variation in early motor and language development. Arch. Dis. Child. 105: 421–427. 31666245 10.1136/archdischild-2019-317849

[35] Tavakol, M. & R. Dennick . 2011. Making sense of Cronbach's alpha. Int. J. Med. Educ. 2: 53–55. 28029643 10.5116/ijme.4dfb.8dfd PMC4205511

[36] Fernald, L.C. , P. Kariger , M. Hidrobo & P.J. Gertler . 2012. Socioeconomic gradients in child development in very young children: evidence from India, Indonesia, Peru, and Senegal. Proc. Natl. Acad. Sci. USA 109: 17273–17280. 23045688 10.1073/pnas.1121241109 PMC3477389

[37] Lung, F.W. , B.C. Shu , T.L. Chiang , *et al*. 2009. Predictive validity of Bayley scale in language development of children at 6–36 months. Pediatr. Int. 51: 666–669. 19419503 10.1111/j.1442-200X.2009.02844.x

[38] Krogh, M.T. & M.S. Væver . 2019. Does gender affect Bayley‐III scores and test‐taking behavior? Infant Behav. Dev. 57. 10.1016/j.infbeh.2019.101352 31445432

[39] Gneezy, U. , K.L. Leonard & J.A. List . 2009. Gender differences in competition: evidence from a matrilineal and a patriarchal society. Econometrica 77: 1637–1664.

[40] Gneezy, U. & A. Rustichini . 2004. Gender and competition at a young age. Am. Econ. Rev. 94: 377–381.

[41] Black, M.M. , K. Bromley , V.A. Cavallera , *et al*. 2019. The Global Scale for Early Development (GSED). Early Child. Matters 14: 80–84.

