# Supplementary figures and images for: Assessing the performance of the Caregiver Reported Early Development Instruments (CREDI) in rural India
Source: Ann N Y Acad Sci. 2020 Dec 30;1492(1):58–72. doi: 10.1111/nyas.14543 (PMC8246540; doi:10.1111/nyas.14543)

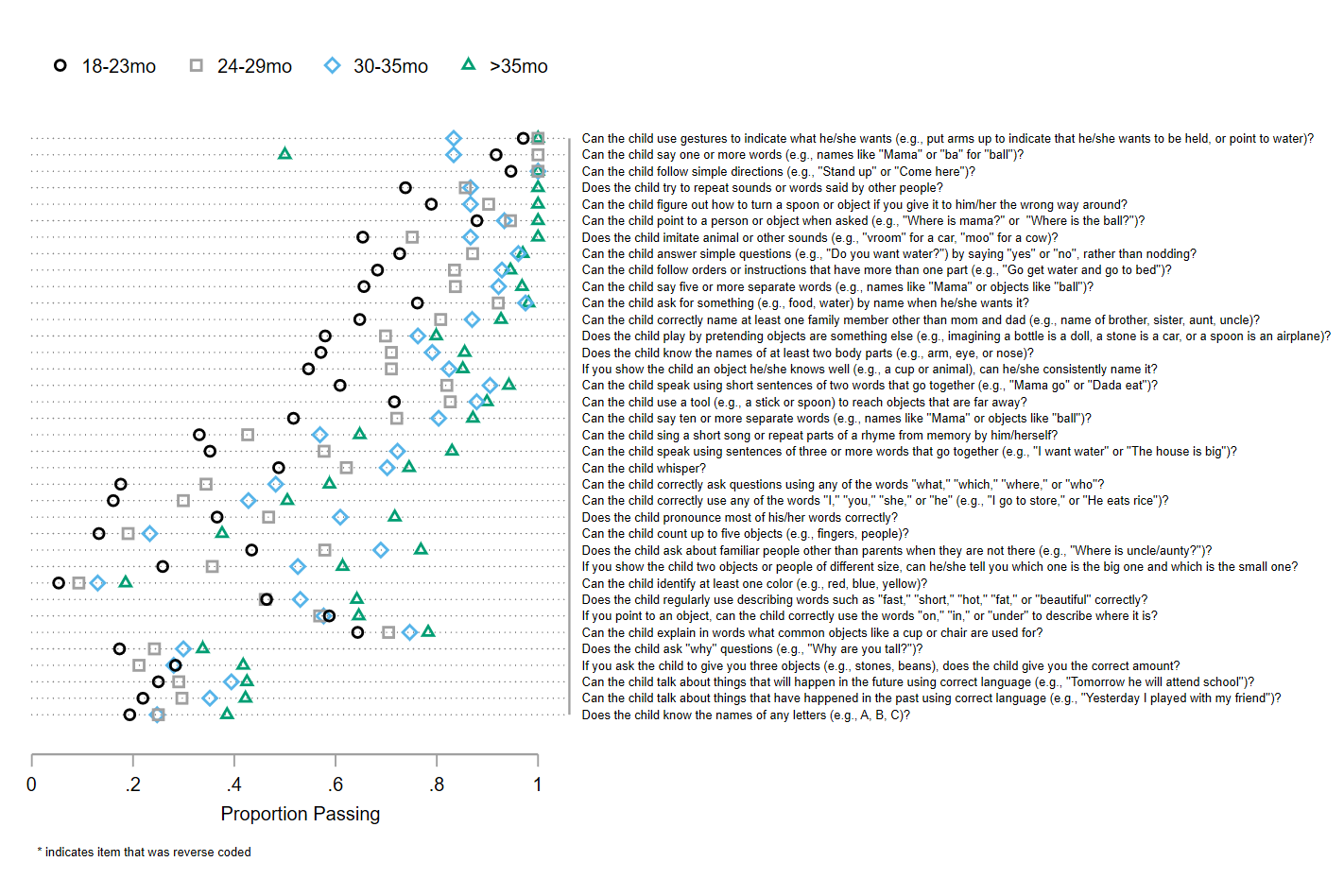

Supplement: Supplementary file 1 — Supplementary Figure S1. [file NYAS-1492-58-s004.tif]

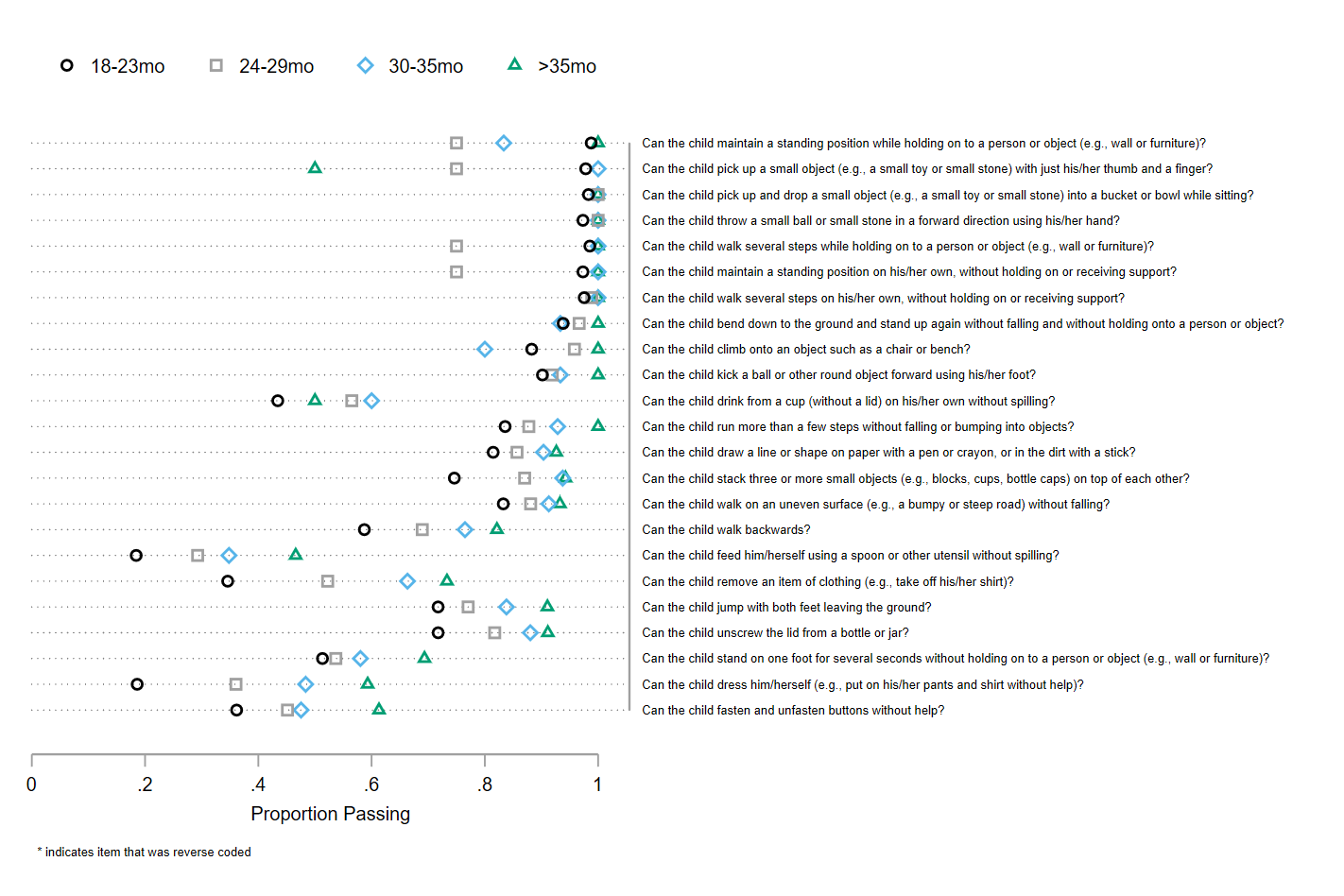

Supplement: Supplementary file 2 — Supplementary Figure S2. [file NYAS-1492-58-s003.tif]

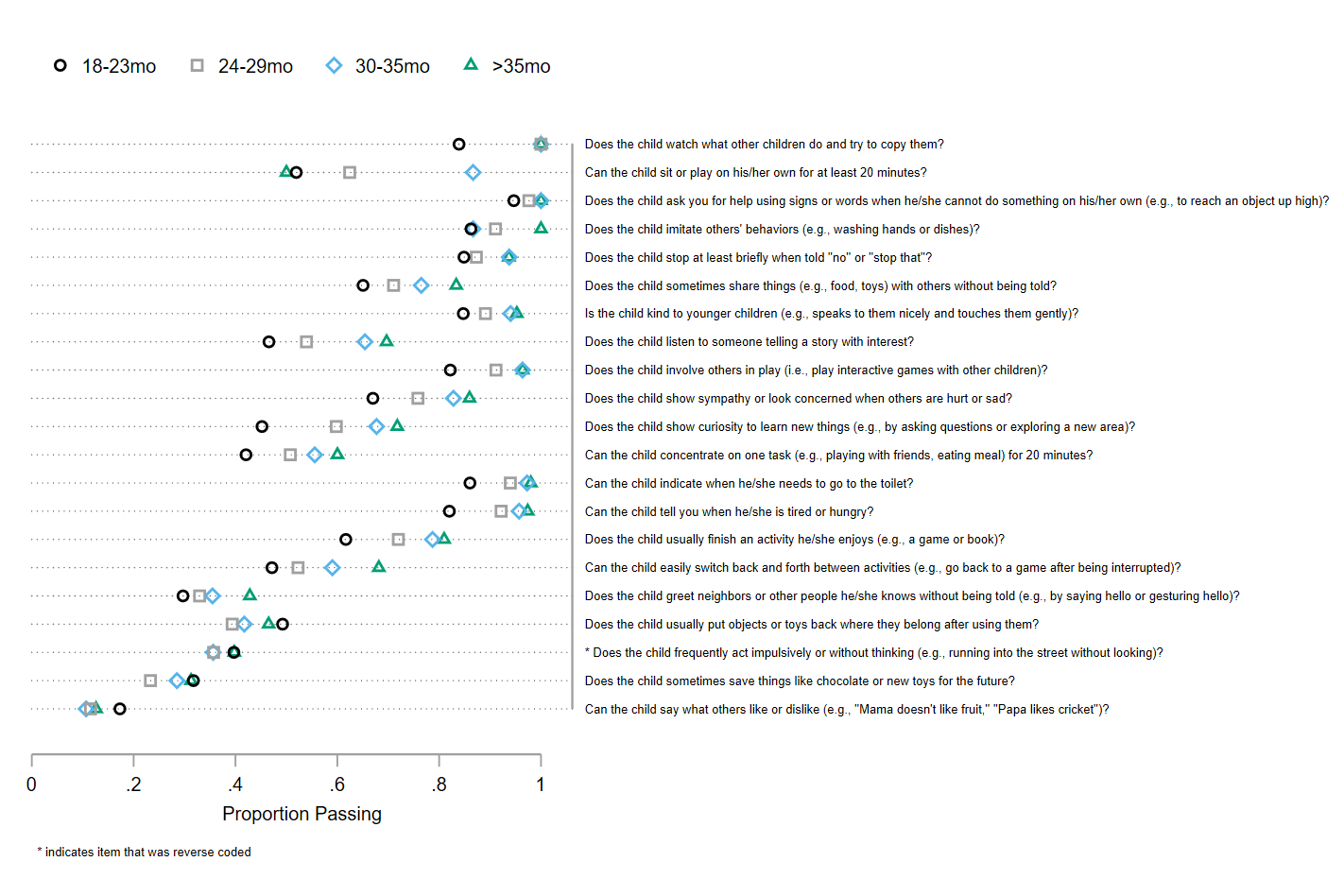

Supplement: Supplementary file 3 — Supplementary Figure S3. [file NYAS-1492-58-s002.tif]
