# Supplementary material for: Assessing the performance of the Caregiver Reported Early Development Instruments (CREDI) in rural India
Source: Ann N Y Acad Sci. 2020 Dec 30;1492(1):58–72. doi: 10.1111/nyas.14543 (PMC8246540; doi:10.1111/nyas.14543)
Supplement: Supplementary file 4 — Supplementary Table S1. Correlations of Bayley‐III Residuals with CREDI Residuals using global normed scores (N = 994) [file NYAS-1492-58-s001.docx]

**Annex 1. Correlations of Bayley-III Residuals with CREDI Residuals using global normed scores (N=994)**

| Bayley-III | CREDI Score | | | | | | | | |  |
| --- | --- | --- | --- | --- | --- | --- | --- | --- | --- | --- |
|  | Interviewer dummies | | | Adding caregiver’s education | | | Adding household socioeconomic quartile | | |  |
|  | Cognitive | Language | Motor | Cognitive | Language | Motor | Cognitive | Language | Motor |  |
| Cognitive | .1937^***^ |  |  | .1623^***^ |  |  | .1234^***^ |  |  |  |
| Language |  | .2960^***^ |  |  | .2665^***^ |  |  | .2054^***^ |  |  |
|  |  |  |  |  |  |  |  |  |  |  |
|  | Mother’s education | | | | | | | | |  |
|  | No education (N=390) | | | Primary education (N=204) | | | Higher than primary education (N=399) | | |  |
|  | Cognitive | Language | Motor | Cognitive | Language | Motor | Cognitive | Language | Motor |  |
| Cognitive | .1168^**^ |  |  | .1361^***^ |  |  | .1482^***^ |  |  |  |
| Language |  | .1994^***^ |  |  | .1796^***^ |  |  | .2021^***^ |  |  |
|  | Maternal depressive symptoms | | | | | |  | | |  |
|  | Low depression (N=824) | | | High depression (N=170) | | |  | | |  |
|  | Cognitive | Language | Motor | Cognitive | Language | Motor |  |  |  |  |
| Cognitive | .1239^***^ |  |  | .1134^***^ |  |  |  |  |  |  |
| Language |  | .2073^***^ |  |  | .2086^***^ |  |  |  |  |  |
|  | By treatment | | | | | |  |  |  |  |
|  | Control (N=496) | | | Treatment (N=497) | | |  |  |  |  |
|  | Cognitive | Language | Motor | Cognitive | Language | Motor |  |  |  |  |
| Cognitive | .1129^***^ |  |  | .1360^***^ |  |  |  |  |  |  |
| Language |  | .1920^***^ |  |  | .2042^***^ |  |  |  |  |  |
|  | By test location | | | | | |  |  |  |  |
|  | Home (N=200) | | | Elsewhere (N=794) | | |  |  |  |  |
|  | Cognitive | Language | Motor | Cognitive | Language | Motor |  |  |  |  |
| Cognitive | .0898^***^ |  |  | .1305^***^ |  |  |  |  |  |  |
| Language |  | .2073^***^ |  |  | .1961^***^ |  |  |  |  |  |

^*^ *p* < 0.10, ^**^ *p* < 0.05, ^***^ *p* < .01.

Note: The table reports the residual correlations of the CREDI and Bayley globally standardized z scores
